# Facile Synthesis of Peptide-Conjugated Gold Nanoclusters with Different Lengths

**DOI:** 10.3390/nano11112932

**Published:** 2021-11-02

**Authors:** Qun Ma, Lichao Liu, Zeyue Yang, Peng Zheng

**Affiliations:** Chemistry and Biomedicine Innovation Center (ChemBIC), State Key Laboratory of Coordination Chemistry, School of Chemistry and Chemical Engineering, Nanjing University, 163 Xianlin Road, Nanjing 210023, China; DG1924063@smail.nju.edu.cn (Q.M.); 2017210058@mail.buct.edu.cn (L.L.); mf20240038@smail.nju.edu.cn (Z.Y.)

**Keywords:** gold nanocluster, peptide conjugation, elastin-like polypeptide, single-molecule force spectroscopy

## Abstract

The synthesis of ultra-small gold nanoclusters (Au NCs) with sizes down to 2 nm has received increasing interest due to their unique optical and electronic properties. Like many peptide-coated gold nanospheres synthesized before, modified gold nanoclusters with peptide conjugation are potentially significant in biomedical and catalytic fields. Here, we explore whether such small-sized gold nanoclusters can be conjugated with peptides also and characterize them using atomic force microscopy. Using a long and flexible elastin-like polypeptide (ELP)_20_ as the conjugated peptide, (ELP)_20_-Au NCs was successfully synthesized via a one-pot synthesis method. The unique optical and electronic properties of gold nanoclusters are still preserved, while a much larger size was obtained as expected due to the peptide conjugation. In addition, a short and rigid peptide (EAAAK)_3_ was conjugated to the gold nanoclusters. Their Yong’s modulus was characterized using atomic force microscopy (AFM). Moreover, the coated peptide on the nanoclusters was pulled using AFM-based single molecule-force spectroscopy (SMFS), showing expected properties as one of the first force spectroscopy experiments on peptide-coated nanoclusters. Our results pave the way for further modification of nanoclusters based on the conjugated peptides and show a new method to characterize these materials using AFM-SMFS.

## 1. Introduction

Gold nanoparticles, particularly gold nanospheres [1,2,3,4,5,6,7,8] (Au NSs, size < 200 nm) and gold nanoclusters [9,10,11,12,13,14,15,16,17] (Au NCs, size < 2 nm), have received growing attention in recent years because of their excellent optical and electronic properties [18,19,20]. Many biomolecule-conjugated Au NSs have been prepared, such as peptide, protein, and DNA, which have a potential use in many biomedical and biosensing applications [21,22,23,24,25,26,27,28,29]. Typically, the peptides are conjugated to the surface of gold nanoparticles through the Au-S bond which was formed between the cysteine and gold [30,31,32,33]. So far, most peptide-conjugated nanoparticles are prepared by using the larger size Au NSs, while the peptide-conjugation of ultra-small Au NCs is relatively less. Considering the much larger size of peptides compared with the nanoclusters, the synthesis of peptide-coated nanoclusters may be complex. Moreover, many protein-conjugated Au NSs have been prepared with more diverse and powerful applications [8,34,35,36,37,38], which are typically directly immobilized by non-specific interactions [39,40]. Here, the conjugation of a lengthy peptide on Au NSs can be the first step toward site-specific protein modification. By conjugating a proper peptide with a recognition site for enzymatic connection, a target protein can be further coated on the nanoclusters via an enzymatic ligation with the peptide, such as using sortase or asparaginyl ligase (AEP) [41,42,43]. Thus, we explore here whether such small-sized Au NCs can be conjugated with a long peptide, with the ultimate goal for site-specific protein immobilization on nanocluster.

**Figure 1:**
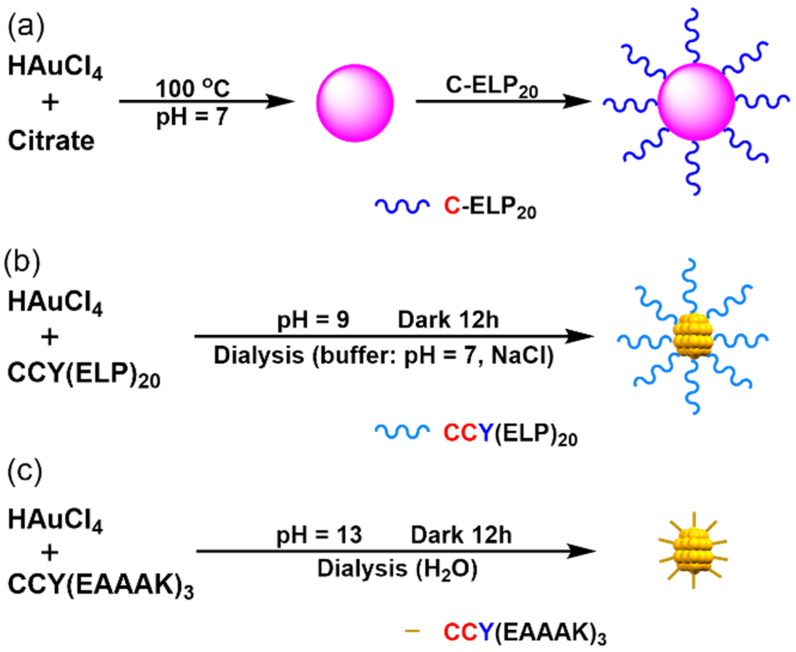
Schematic illustration of the synthesis of (**a**) (ELP)_20_-Au NSs. (**b**) (ELP)_20_-Au NCs. (**c**) (EAAAK)_3_-Au NCs.

Tow peptides with different lengths were used for conjugation (Figure 1). First, a long and flexible elastin-like polypeptide (ELP), consisting of a repeat unit of Val-Pro-Gly-Xaa-Gly derived from human tropoelastin, was selected. Due to its unique thermos-responsive ability and low-mechanical strength, the ELP fragment has been used to synthesize smart-nanoparticle responsible for the change of temperature and be used as a soft linker for single-molecule studies [42,44,45,46,47,48,49,50]. Specifically, a polypeptide of twenty ELP units, (ELP)_20_, was used here, which has nearly twenty times higher length than the nanocluster (~20 nm vs. 1 nm) with a high molecular weight (~10 kDa). Secondly, a rigid peptide (EAAAK)_3_ with a comparable length (~7.5 nm) as the nanoclusters was tested. Moreover, it is worth noting that (EAAAK)_3_ has been widely used as the scaffold in biomacromolecule conjugation [51].

## 2. Materials and Methods

### 2.1. Materials

Hydrogen tetrachloroaurate (III) trihydrate (HAuCl_4_·3H_2_O) was purchased from Alfa Aesar (Shanghai, China). Trisodium citrate dihydrate was purchased from Sinopharm Chemical Reagent Co. Ltd. (Shanghai, China). Peptide CCY(EAAAK)_3_ was synthesized using a solid-phase method (Sangon Biotech Co. Ltd., purity 95%) (Shanghai, China). Other reagents were purchased from Sangon Biotech Co. Ltd. (Shanghai, China). All reagents were used without further purification. Ultrapure water (18 MΩ cm^−1^) was obtained from a Millipore Milli-Q Advantage water purification system (Burlington, MA, USA). *E. coil* BL21 (DE3) and XL1-Blue cells were purchased from TransGen Biotech Co. Ltd. (Beijing, China). The glass coverslips were purchased from Sail Brand, China. The AFM cantilevers (MLCT-Bio-DC) were purchased from Bruker Corp (Billerica, MA, USA).

### 2.2. Sample Characterizations

The size and morphology of nanoparticles and clusters were observed using a JEOL JEM-2100 transmission electron microscope (Tokyo, Japan) at 200 kV and a Thermo Scientific Talos L120C TEM (Waltham, MA, USA) at 120 kV. Dynamic light scattering (DLS) was recorded at a wavelength of 659 nm and zeta potential experiments were conducted on 90 Plus/BI-MAS equipment (Brookhaven, NY, USA). The concentration of Au was determined by inductively coupled plasma mass spectrometry (Thermo X Series 2 ICP-MS, Waltham, MA, USA). The UV-Vis spectra were measured on an Ocean Optics Maya 2000 Pro spectrometer (Orlando, FL, USA). The fluorescence spectra were measured on a HORIBA Jobin Yvon Fluoromax-4 fluorescence spectrometer (Irvine, CA, USA). The concentrations of CCY(ELP)_20_ and C-(ELP)_20_ were determined using the Ellman method. The AFM experiments were performed on a Nanowizard4 AFM (JPK, Berlin, Germany). Mass spectrometric analyses were performed using an UltrafleXtreme MALDI-TOF mass spectrometer (Bruker Daltonics, Billerica, MA, USA) operating in linear positive ion mode with 2,5-Dihydroxybenzoic acid (DHB) as the matrix.

### 2.3. Protein Engineering

ELP is the abbreviation of the elastin-like polypeptide. The genes of (ELP)_20_ were purchased from Genscript (Nanjing, China). Construction of C-(ELP)_20_ and CCY(ELP)_20_ was in the expression vector pET-28a by standard molecular biology and PCR techniques.

The plasmids were transformed and then overexpressed in *E. coli* BL21 (DE3) cells. The bacteria kept in the LB medium containing 50 µg mL^−1^ kanamycin were grown to an OD_600_ = 0.6 and then were induced by 0.5 mM isopropyl β-D-thiogalactoside (IPTG) overnight at 18 °C. The cells were obtained by centrifugation at 9000 rpm for 5 min at 4 °C (Avanti JXN series, Beckman Coulter, Brea, CA, USA). The cells were redispersed in buffer (50 mM Tris, 100 mM NaCl, pH 7.0) and lysed via a high-pressure homogenization. The supernatants were mixed with Co-NTA affinity beads and then kept for 2 to 3 h after the centrifugation operation at 20,000 rpm for 30 min. The mixture was washed with buffer (20 mM Tris, 400 mM NaCl, 2 mM imidazole, pH 7.0) several times and eluted in buffer (20 mM Tris, 400 mM NaCl, 250 mM imidazole, pH 7.0) immediately.

### 2.4. Synthesis of (ELP)_20_-Au Nanospheres

Citrate capped Au nanospheres were synthesized according to a previously published method [52]. Briefly, HAuCl_4_ (1 mL, 25 mM) was injected into a boiling aqueous solution of sodium citrate (2.2 mM, 150 mL) under magnetic stirring. The solution was cooled to room temperature when the solution turned red. The concentration of Au (0.18 mM) was determined by ICP-MS.

1.5 mL of C-(ELP)_20_ (1.3 µmoL) solution was added into the 20 mL Au-Cit solution dropwise [53]. The sample was stirred for three hours at room temperature. The product (ELP)_20_-Au NSs were collected by centrifugation and washed with buffer (50 mM Tris, 100 mM NaCl, pH 7.0) several times. Finally, the (ELP)_20_-Au NSs were dispersed in buffer (50 mM Tris, 100 mM NaCl, pH 7.0). Based on the quantity of the reactant HAuCl_4_ and peptide, we estimated the concentration of NSs is 5 × 10^4^ ppm with a cove rate of 5.

### 2.5. Synthesis of (ELP)_20_-Au Nanoclusters

First, CCY(ELP)_20_ was obtained from C-(ELP)_20_ using the standard molecular biology method. Then, (ELP)_20_-AuNCs were synthesized accordingly to the previously reported method. In a typical experiment, an aqueous solution of HAuCl_4_ (70 µL, 25 mM) was added slowly to the CCY(ELP)_20_ solution (1.7 mL, 1 mM) under vigorous stirring in a 5 mL flask. Then NaOH solution (300 µL, 0.5 M) was added to adjust the pH of the sample to 9. The mixture was reacted for 12 h in the dark. Finally, the product (ELP)_20_-Au NCs were purified by dialysis (Dialysis Membrane MWCO: 100 KD) against buffer (50 mM Tris, 100 mM NaCl, pH 7.0) for three days. Similarly, we estimated the concentration of NCs is 77.5 ppm with a cove rate of 37.

### 2.6. Synthesis of (EAAAK)_3_-Au Nanoclusters

The synthesis of (EAAAK)_3_-Au NCs was similar to (ELP)_20_-Au NCs. First, 3.4 mg peptide CCY(EAAAK)_3_ was dissolved in 1.65 mL H_2_O. Next, an aqueous solution of HAuCl_4_ (66 µL, 25 mM) was added slowly to the CCY(EAAAK)_3_ solution (1 mM) under vigorous magnetic stirring in a 5 mL flask. Then NaOH solution (300 µL, 0.5 M) was added to adjust the pH of the sample to 13. The mixture was reacted for 12 h in the dark. Finally, the product (EAAAK)_3_-Au NCs were purified by dialysis (Dialysis Membrane MWCO: 10 KD) against H_2_O for three days. We estimated the concentration of NCs is 116 ppm with a cover rate of 38.

## 3. Results and Discussions

### 3.1. Synthesis and Characterization of Au-Cit and (ELP)_20_-Au NSs

**Figure 2:**
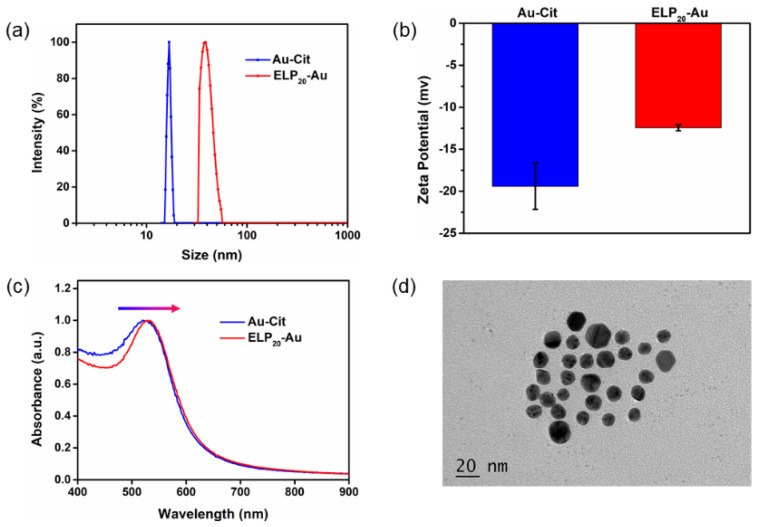
Characterizations of synthesized Au-Cit and (ELP)_20_-Au NSs. (**a**) Hydrodynamic diameter distribution (**b**) Zeta potential (**c**) UV-Vis spectra of Au-Cit and (ELP)_20_-Au NSs. (**d**) Transmission electron microscopy image of (ELP)_20_-Au NSs.

To better demonstrate the synthesis and property of the (ELP)_20_-conjugated gold nanoclusters, we synthesized the (ELP)_20_-gold nanospheres with a larger particle size first for comparison. The Au-Cit NSs were synthesized by the classic method described above. The TEM picture (Figure S1) showed that the Au-Cit NSs were monodispersed in water with an average diameter of 9 nm. Peptide C-(ELP)_20_ was naturally produced using *E. coli* expression. The purified peptide showed an expected molecular weight of ~10 kDa, verified by SDS-PAGE gel (Figure S2). Then the (ELP)_20_-Au NSs were synthesized by the ligand-exchange method owing to the Au-S bond formation between cysteine residues in C-(ELP)_20_ and Au-Cit NSs (Figure 1a). Dynamic light scattering (DLS) and zeta potential experiments were conducted to further prove its peptide conjugation on the nanospheres. The changes of hydrodynamic diameter distribution along with Zeta potential indicated that the conjugation of flexible peptide C-(ELP)_20_ on the NSs is successful (Figure 2a,b). In addition, the UV-Vis absorption spectroscopy showed that the surface plasmon resonance peak is red-shifted from 520 to 530 nm after being modified with peptide C-(ELP)_20_ (Figure 2c). The modified (ELP)_20_-Au NSs were well monodisperse without aggregation in buffer (50 mM Tris, 100 mM NaCl, pH 7.0) as shown in the TEM picture (Figure 2d). Furthermore, the morphology of the prepared (ELP)_20_-Au NSs experienced no change at all compared with the Au-Cit NSs (Figure S1).

### 3.2. Synthesis and Characterization of (ELP)_20_-Au NCs

**Figure 3:**
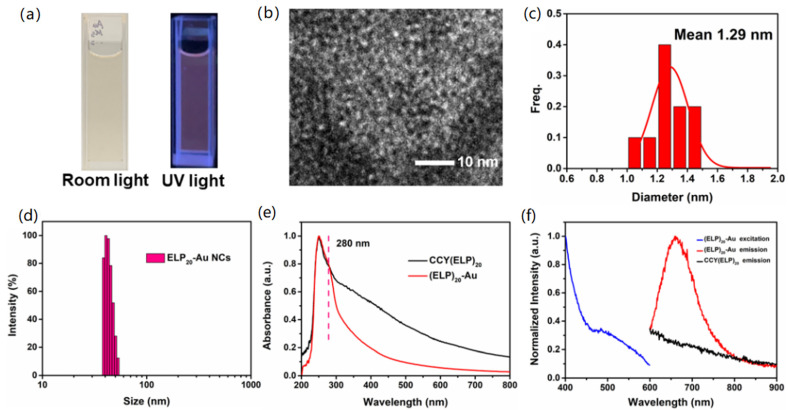
Characterizations of (ELP)_20_-Au NCs. (**a**) Photographs of (ELP)_20_-Au NCs under room light and UV light. (**b**) Negative-stain EM image of the (ELP)_20_-Au NCs. (**c**) The histogram of diameter distribution of the (ELP)_20_-Au NCs. (**d**) Hydrodynamic diameter distribution of the (ELP)_20_-Au NCs. (**e**) UV-Vis spectra of CCY(ELP)_20_ and (ELP)_20_-Au NCs. (**f**) Fluorescence excitation (blue) and emission spectra of (ELP)_20_-Au NCs (red) and CCY(ELP)_20_ (black).

Next, we explore whether the ultra-small Au nanocluster conjugated with the long peptide, CCY(ELP)_20_, can also be synthesized (Figure 1b, Figure S3 and Figure S4). Here, the peptide-conjugated gold nanoclusters were synthesized using a one-pot method, in which the formation of gold nanoclusters and the peptide conjugation were achieved at the same step. In short, the tyrosine in the CCY(ELP)_20_ functioned as a reducing agent and participated in the formation of (ELP)_20_-Au NCs [10,54]. Thus, the ELP peptide was conjugated in the same step. The details for the synthesis can be found above. As shown in Figure 3a, the solution of (ELP)_20_-Au NCs exhibited light yellow under room light and pink fluorescence under UV light, consistent with the formation of gold nanoclusters. Compared to Au NSs, luminescence is the unique property of Au nanoclusters. It is believed that the small size of the Au NSs (2 nm) is comparable to Fermi wavelength of electrons (<1 nm) and thus has a visible-to-near infrared fluorescence. It indicates the unique optical property of Au NCs is preserved after the conjugation of a long peptide. The negative-stain EM image and the histogram revealed the Au NCs with an average diameter of 1.29 nm (Figure 3b,c). In addition, the aggregation state of CCY(ELP)_20_ from the negative-stain EM image also confirmed the successful synthesis of the nanoclusters (Figure S5). Moreover, DLS results of (ELP)_20_-Au NCs (Figure 3d) showed that the hydrodynamic diameter was about 40 nm, much larger than the size observed with TEM. It agrees well with a long ELP peptide’s conjugation and proves the CCY(ELP)_20_ conjugation on the nanocluster. The (ELP)_20_-Au NCs showed a broad absorption band ranging from 250 nm to 800 nm with a small absorption peak located at 280 nm, which can be attributed to the absorption of tyrosine. The location of this peak and the absorption spectrum matched with CCY(ELP)_20_ (Figure 3e). The fluorescence excitation and emission spectra of CCY(ELP)_20_ and (ELP)_20_-Au NCs were depicted in Figure 3f. The fluorescence emission spectra of (ELP)_20_-Au NCs ranged from 600 to 800 nm, with the emission peak locating at 659 nm when excited at 250 nm with the insertion of 300 nm optical filter in the excitation window. At the same time, the CCY(ELP)_20_ exhibited almost negligible emission. In summary, all data proved that the (ELP)_20_-Au NCs are successfully synthesized. Moreover, the composition of the (ELP)_20_-Au NCs were characterized by using matrix-assisted laser desorption/ionization time of flight mass spectrometry (MALDI-TOF MS). The mass spectrum indicated that (ELP)_20_-Au NCs were mainly composed of Au_21_ and Au_23_ (Figure S6).

### 3.3. Synthesis and Characterization of (EAAAK)_3_-Au Nanoclusters

**Figure 4:**
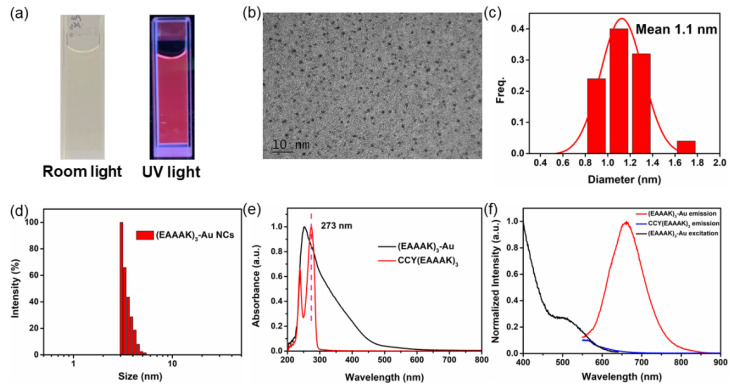
Characterizations of prepared (EAAAK)_3_-Au NCs. (**a**) Photographs of (EAAAK)_3_-Au NCs under room light and UV light. (**b**) TEM image of the (EAAAK)_3_-Au NCs. (**c**) The diameter distribution histogram of the (EAAAK)_3_-Au NCs. (**d**) Hydrodynamic diameter distribution of the (EAAAK)_3_-Au NCs. (**e**) UV-Vis spectra of CCY(EAAAK)_3_ and (EAAAK)_3_-Au NCs. (**f**) Fluorescence excitation (black) and emission spectra of the (EAAAK)_3_-Au NCs (red) and CCY(EAAAK)_3_ (blue).

In addition to the synthesis of a long (ELP)_20_-conjugated gold nanoclusters, we demonstrate the synthesis of another short peptide CCY(EAAAK)_3_ -conjugated nanoclusters (Figure 1c and Figure S7). The synthesis procedure is almost the same as the previous one, which can be found in Section 2.6. As shown in Figure 4a, the solution of (EAAAK)_3_-Au NCs appeared light brown under room light and pink fluorescence under UV light. TEM images showed the morphology and monodispersity of (EAAAK)_3_-Au NCs with an average diameter of 1.1 nm (Figure 4b,c and Figure S8). It is noted that the peptide was not detectable in TEM, and the value here reflects the diameter of the Au nanocluster core. Indeed, DLS results of (EAAAK)_3_-Au NCs (Figure 4d) showed an average diameter of ~3 nm (Raw data in Table S1). Nevertheless, the detection limit of our DLS is 2 nm. Thus, the actual diameter may be biased and smaller than the apparent DLS result. The (EAAAK)_3_-Au NCs showed a broad absorption band ranging from 250 nm to 800 nm with a small absorption peak located at 273 nm, which is the characteristic absorption of tyrosine. The location of this peak and the absorption spectrum fitted with CCY(EAAAK)_3_ (Figure 4e). Figure 4f showed the fluorescence excitation and emission spectra of CCY(EAAAK)_3_ and (EAAAK)_3_-Au NCs. The fluorescence emission spectra of (EAAAK)_3_-Au NCs ranged from 550 to 850 nm, with the emission peak at 660 nm when excited at 498 nm, similar to the preceding experimental results. The emission spectra of (EAAAK)_3_-Au NCs were shown in Figure S9 and the peak of emission spectrum peak was at 660 nm. Furthermore, the mass spectrum showed that (EAAAK)_3_-Au NCs mainly consisted of Au_16_ and Au_21_ (Figure S10).

### 3.4. Characterization of Peptide-Modified Nanocluster by Atomic Force Microscopy

**Figure 5:**
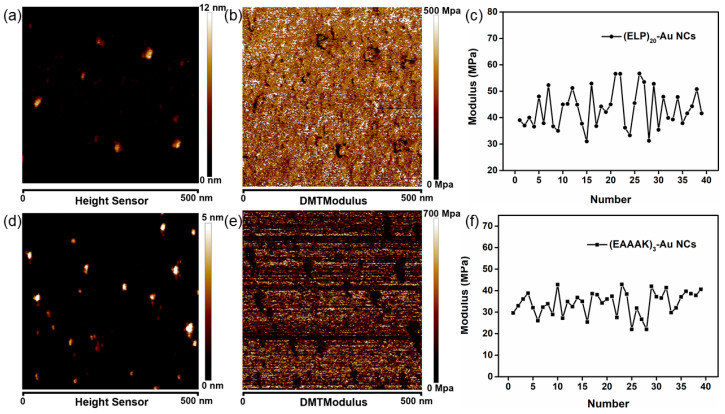
AFM imaging and Young’s modulus measurement of the Au NCs. (**a**) AFM image (**b**) Young’s modulus measurement and (**c**) distribution of (ELP)_20_-Au NCs. (**d**) AFM image (**e**) Young’s modulus measurement and (**f**) distribution of (EAAAK)_3_-Au NCs.

To verify the differences between the two types of peptides-modified Au NCs, atomic force microscopy imaging and Young’s modulus measurement experiments were conducted. Briefly, the Au NCs were dispersed on the mica evenly and immersed in the corresponding buffer. The Au NCs on the mica showed a distinct difference in the morphology and Young’s modulus (Figure 5) when compared with the substrate mica (Figure S11). In addition, (ELP)_20_-Au NCs appeared larger in size and irregular in morphology in contrast with (EAAAK)_3_-Au NCs (Figure 5a,d), which is possibly attributed to the larger molecular weight of CCY(ELP)_20_ than CCY(EAAAK)_3_. It is worth noting that the brighter particle in Figure 5d is the aggregation of the (EAAAK)_3_-Au NCs, which is caused by the addition of the adsorption buffer (KCl), which helps the adsorption of (EAAAK)_3_-Au NCs on the mica. Moreover, Young’s modulus of (ELP)_20_-Au NCs measured is about 50 MPa, which is higher than the (EAAAK)_3_-Au NCs (~35 MPa) (Figure 5).

**Figure 6:**
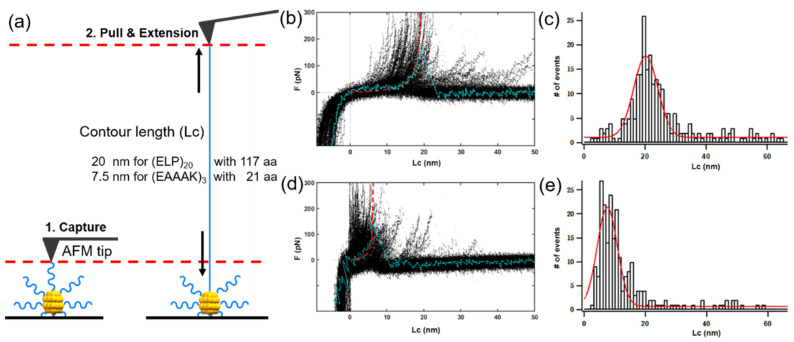
AFM-based single-molecule force spectroscopy (AFM-SMFS) pulling experiments. (**a**) Schematic illustration of pulling the peptide conjugated Au NCs. (**b**) The overlay of all pulling curves of peptide CCY(ELP)_20_ coated on the (ELP)_20_-Au NCs. A representative curve is colored in blue. (**c**) The distribution of contour length (Lc) of (ELP)_20_-Au NCs. (**d**) The overlay of all pulling curves from peptide CCY(EAAAK)_3_ coated on the (EAAAK)_3_-Au NCs. (**e**) The distribution of Lc of (EAAAK)_3_-Au NCs.

AFM imaging showed that the Au NCs were dispersed uniformly and in the state of a single nanoparticle (Figure 5a,d). Based on this, we conducted the atomic force microscopy-based single-molecule force spectroscopy (AFM-SMFS) experiment to pull the coated peptide (Figure 6a). First, the AFM tip approached and captured the peptide. Then the tip pulled the peptide vertically, leading to a length increment from the peptide extension. Finally, the peptide detached from the tip, and the detachment force was recorded. All corresponding force-extension curves on the (ELP)_20_-Au NCs (Figure 6b), and (EAAAK)_3_-Au NCs (Figure 6d) were shown and fitted by the worm-like chain model describing the elasticity of peptide/protein polymer (red dash line). A representative curve was highlighted and colored in cyan. Their statistic of the contour length showed an average contour length of 20 nm and 7.5 nm for the two clusters (Figure 6c for (ELP)_20_, Figure 6e for (EAAAK)_3_, respectively. Interestingly, the values are approximately equal to the length of all amino acids stretched completely in the peptide.

## 4. Conclusions

In this work, we demonstrate the conjugation of a long and flexible peptide CCY-(ELP)_20_ on an ultra-small gold nanocluster via the one-pot synthesis. The nanocluster remains its unique optical property while the hydrodynamic diameter increases significantly due to the conjugated long peptide. Also, a short and rigid peptide-conjugated gold nanocluster is synthesized with the expected property, characterized by classic method and atomic force microscopy. Thus, we believe gold nanocluster is suitable for conjugation/functionalization with most peptide sequences, regardless of their size and length. And many previous works on peptide-conjugated nanoparticles can be adopted for nanocluster and may show better performance due to the smaller nanocluster size.

In addition, the conjugated peptide may modify the properties of AuNCs. For example, the coated long peptide (ELP)_20_ may increase the contact area between the AuNCs and the surface, leading to increased friction and a modification of the tribological properties of the nanocluster. And the coated peptide may change the optical properties of the AuNCs if the peptide possesses additional optical properties.

Moreover, the demonstration here may have great potentials for further modification of gold nanoclusters. Enzymes can recognize many specific peptide sequences for ligation, such as sortase and asparaginyl ligase OaAEP1 [42,43,55,56,57], and the use of a suitable peptide as a linker for protein immobilization may allow further characterization by single-molecule force spectroscopy [58,59,60,61,62,63,64,65,66], which may provide mechanical information about the nanocluster and immobilized protein [67,68,69,70,71]. Thus, by designing a proper peptide sequence for a first step peptide-conjugation, further functionalization and characterization of the nanoclusters can be possible [72,73,74,75,76,77].

## Data Availability

Not applicable.

## Supplementary Materials

The following are available online at https://www.mdpi.com/article/10.3390/nano11112932/s1, Table S1. Raw data of hydrodynamic diameter distribution of (EAAAK)_3_-Au NCs measured by DLS. Figure S1. TEM image of Au-Cit NSs. Figure S2. SDS-PAGE result of C-ELP_20_. Figure S3. SDS-PAGE result of CCY(ELP)_20_. Figure S4. MALDI-TOF MS result of CCY(ELP)_20_. Figure S5. Low-magnified negative-stain EM image of (ELP)_20_-Au NCs. Figure S6. MALDI TOF-MS result of (ELP)_20_-Au NCs. Figure S7. MALDI-TOF MS result of CCY(EAAAK)_3_. Figure S8. TEM image of (EAAAK)_3_-Au NCs. Figure S9. The emission spectra of (EAAAK)_3_-Au NCs. Figure S10. MALDI TOF-MS result of (EAAAK)_3_-Au NCs. Figure S11. AFM imaging and Young’s modulus measurement of mica substrate.
