# Supplementary material for: Facile Synthesis of Peptide-Conjugated Gold Nanoclusters with Different Lengths
Source: Nanomaterials (Basel). 2021 Nov 2;11(11):2932. doi: 10.3390/nano11112932 (PMC8623805; doi:10.3390/nano11112932)
Supplement: Supplementary file 1 [file nanomaterials-11-02932-s001.zip › nanomaterials-1430277-supplementary.pdf]

Supplementary Information

Facile Synthesis of Peptide-Conjugated Gold Nanoclusters with Different Lengths

Qun Ma, Lichao Liu, Zeyue Yang and Peng Zheng *

Chemistry and Biomedicine Innovation Center (ChemBIC), State Key Laboratory of Coordination Chemistry, School of Chemistry and Chemical Engineering, Nanjing University, 163 Xianlin Road, Nanjing 210023, China; DG1924063@smail.nju.edu.cn (Q.M.); 2017210058@mail.buct.edu.cn (L.L.); mf20240038@smail.nju.edu.cn (Z.Y.)

* Correspondence: pengz@nju.edu.cn

Supplementary Notes

Peptide sequences

C-ELP$_{20}$ (MW: ~10 *k*Da)

MGHHHHHHCGSVPGEGVPGVGVPGVGVPGVGVPGVGVPGAGVPGAGVPGGGVPGGGVPGEGVPGEGVPGVGVPGVGVPGVGVPGVGVPGAGVPGAGVPGGGVPGGGVPGEGRSNGL

CCY(ELP)$_{20}$ (MW: ~10 *k*Da)

MGHHHHHHCCYGSVPGEGVPGVGVPGVGVPGVGVPGVGVPGAGVPGAGVPGGGVPGGGVPGEGVPGEGVPGVGVPGVGVPGVGVPGVGVPGAGVPGAGVPGGGVPGGGVPGEGRSNGL

CCY(EAAAK)$_{3}$ (MW: 2083 Da):

CCYEAAAKEAAAKEAAAKNGL

Table S1. Raw data of hydrodynamic diameter distribution of $(EAAAK)_3$-Au NCs measured by DLS.

D (nm)	Intensity
2.24	0.00
2.42	0.00
2.62	0.00
2.83	0.00
3.06	100.00
3.31	65.96
3.57	43.51
3.86	28.70
4.18	18.73
4.52	7.99
4.88	2.53
5.28	1.58
5.71	0.00
6.17	0.00
6.67	0.00
7.21	0.00

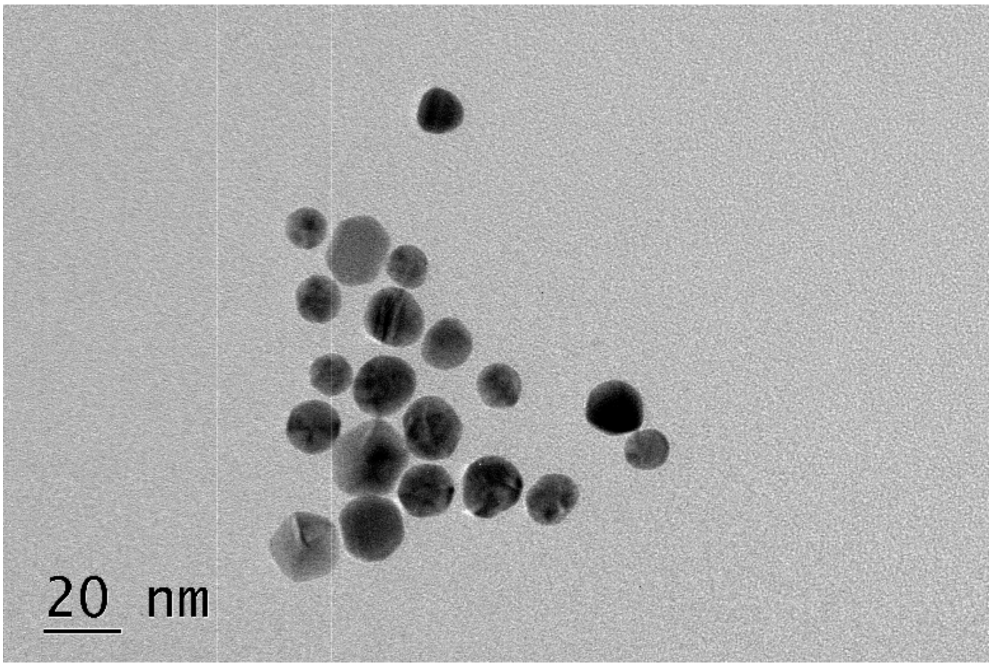

Figure S1. TEM image of Au-Cit NSs.

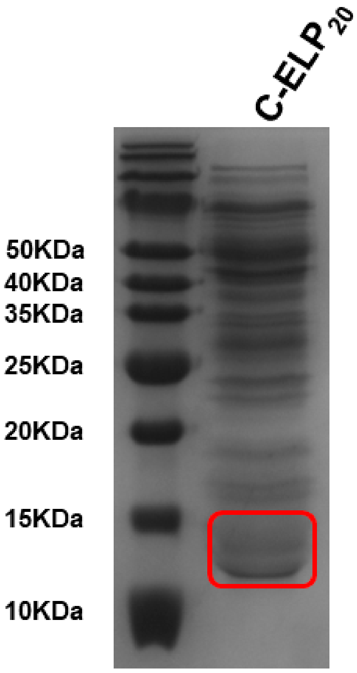

Figure S2. SDS-PAGE result of C-ELP20.

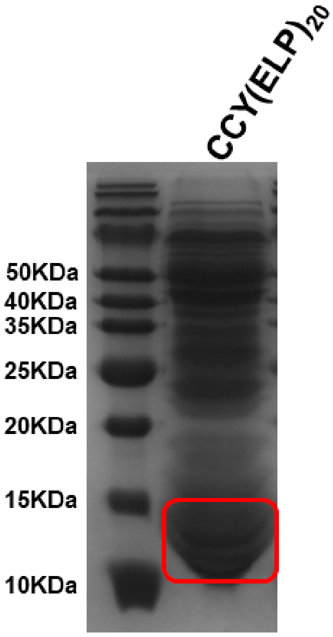

Figure S3. SDS-PAGE result of CCY(ELP)20.

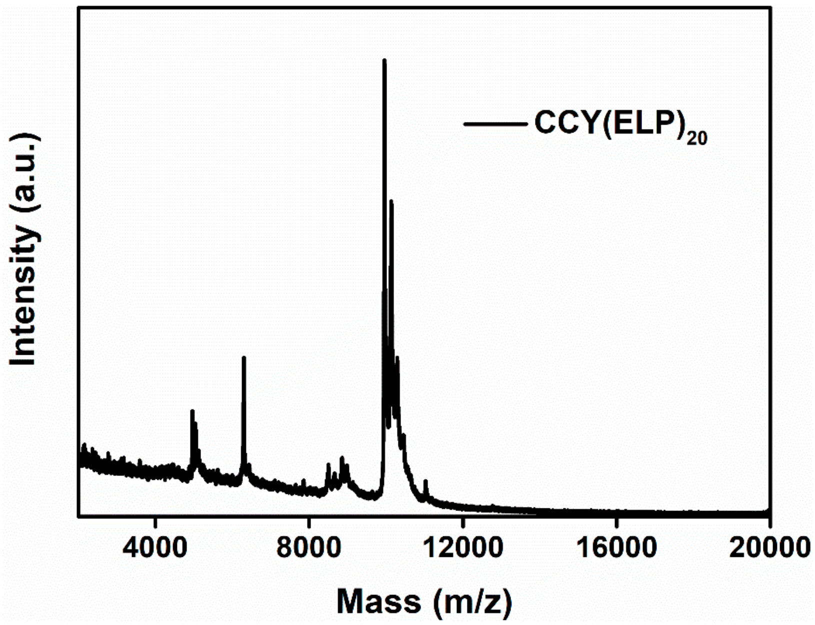

Figure S4. MALDI-TOF MS result of CCY(ELP)20.

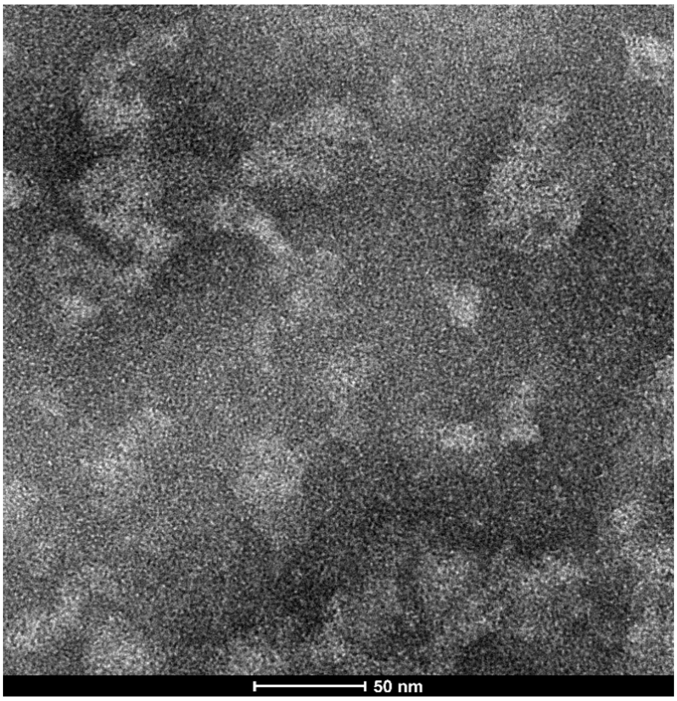

Figure S5. Low-magnified negative-stain EM image of (ELP)20-Au NCs.

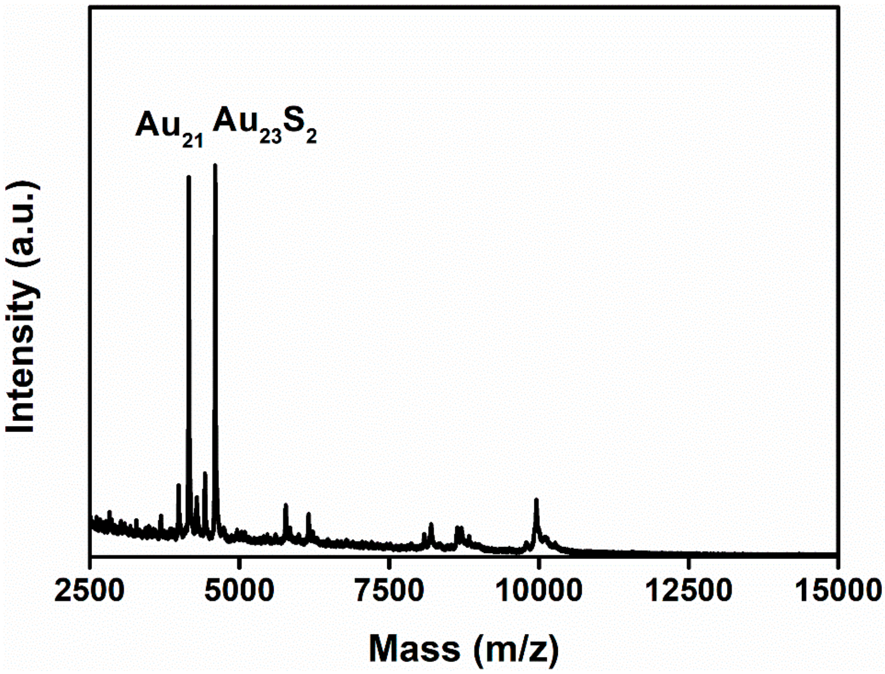

Figure S6. MALDI TOF-MS result of (ELP)20-Au NCs.

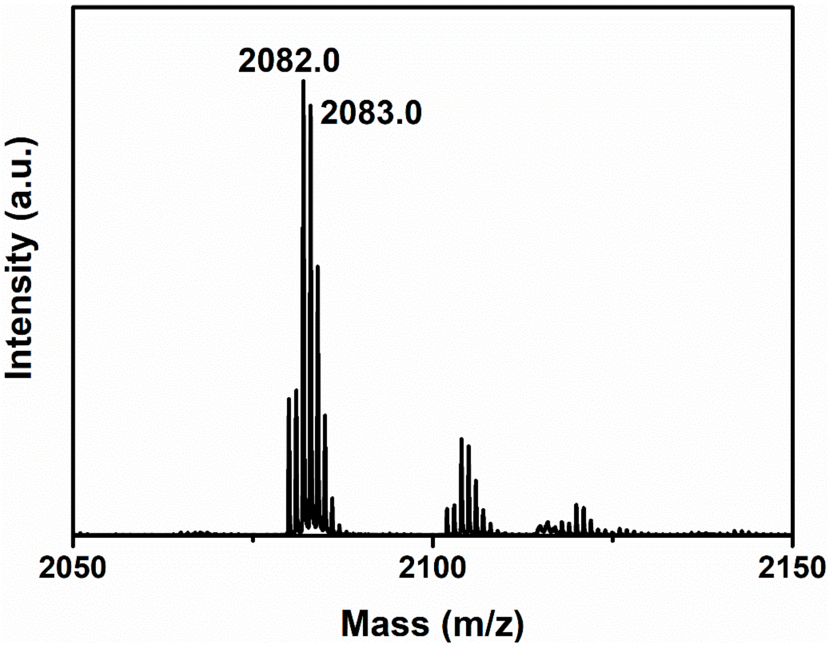

Figure S7. MALDI-TOF MS result of CCY(EAAAK)3.

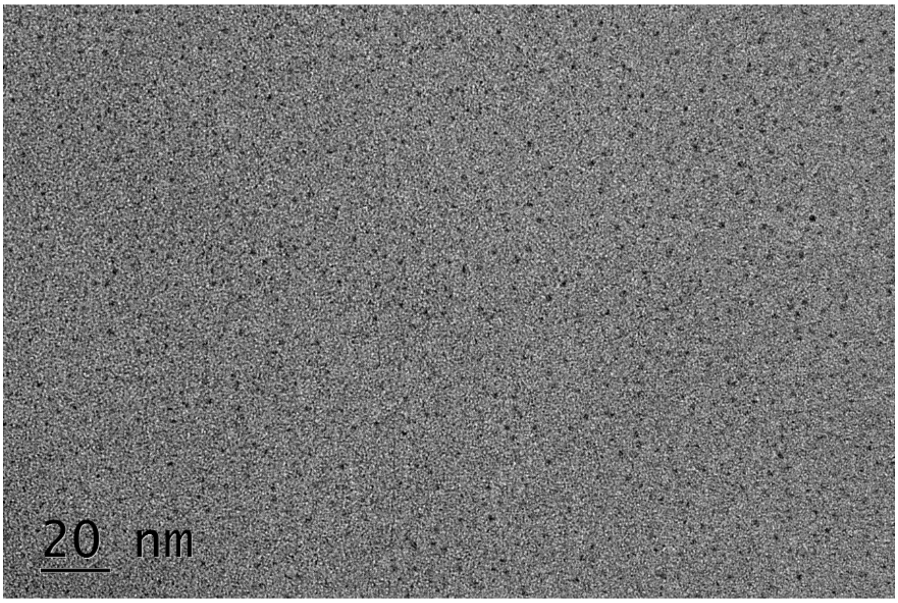

Figure S8. TEM image of (EAAAK)3-Au NCs.

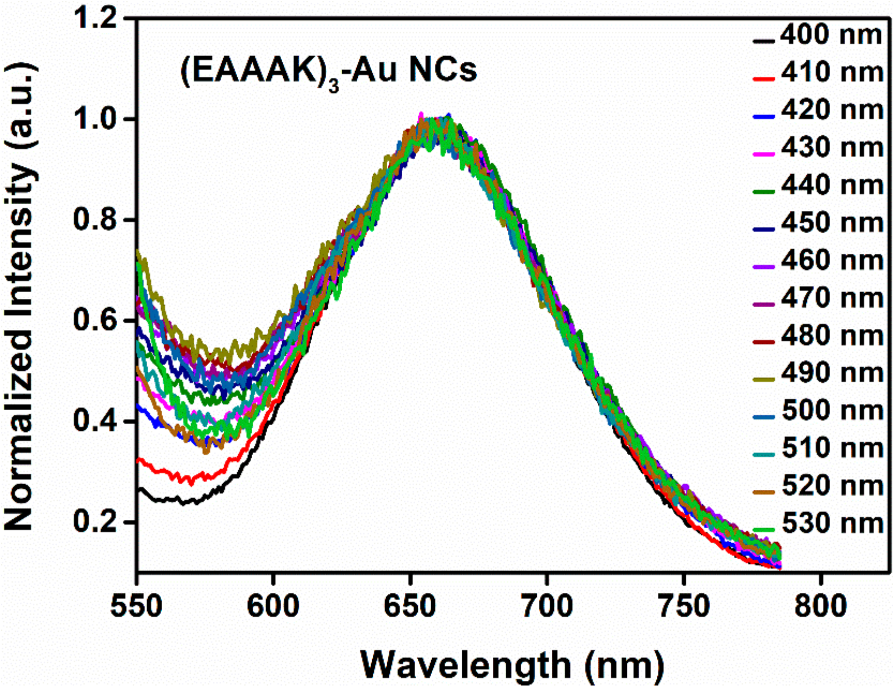

Figure S9. The emission spectra of (EAAAK)3-Au NCs.

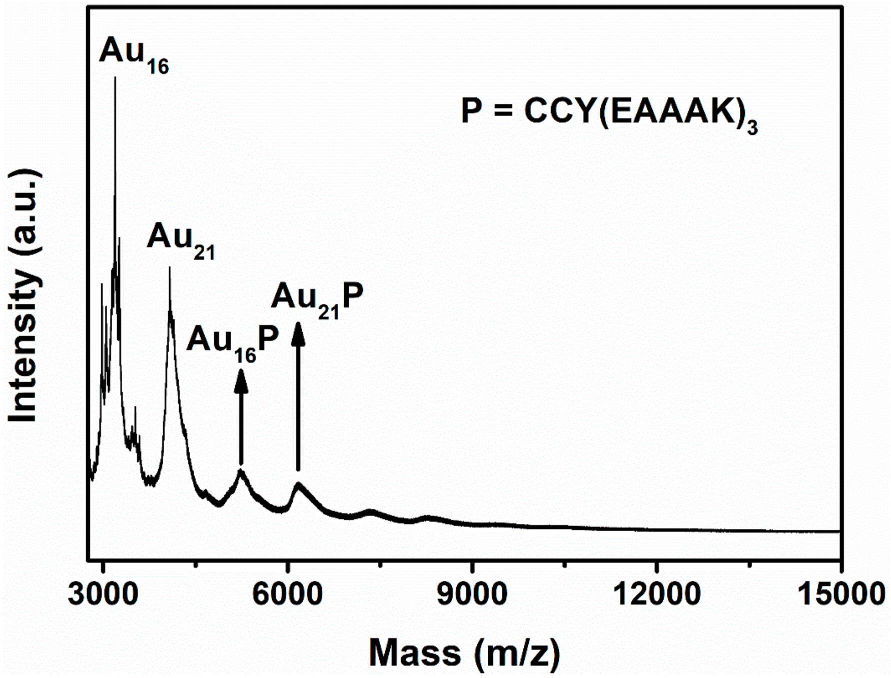

Figure S10. MALDI TOF-MS result of (EAAAK)3-Au NCs.

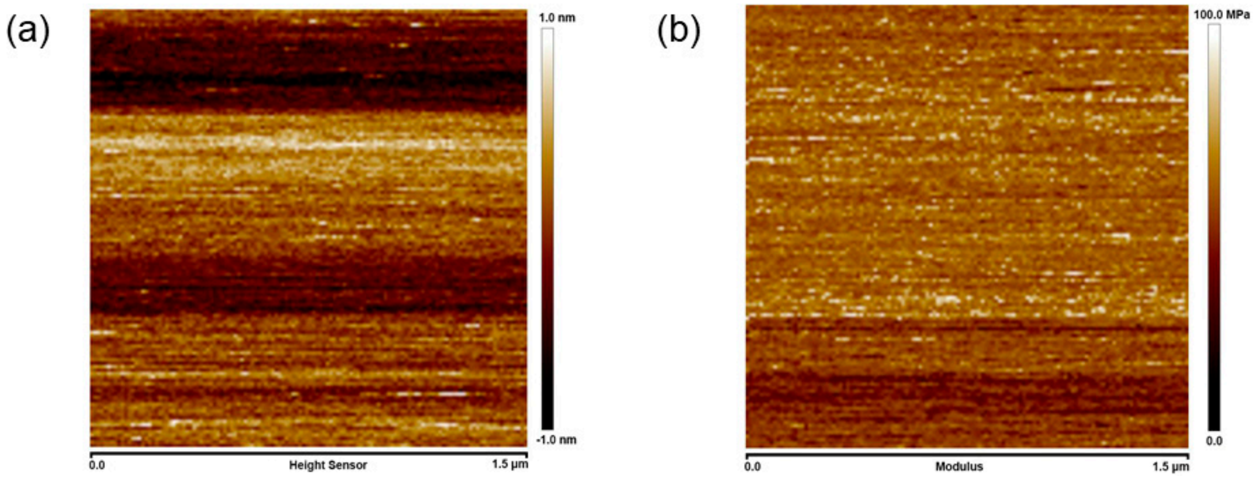

Figure S11. (**a**) AFM imaging and (**b**) Young's modulus measurement of mica substrate.
